# Protective Places: the Relationship between Neighborhood Quality and Preterm Births to Black Women in Oakland, California (2007–2011)

**DOI:** 10.1007/s11524-022-00624-8

**Published:** 2022-04-06

**Authors:** Rachel L. Berkowitz, Mahasin Mujahid, Michelle Pearl, Victor Poon, Carolina K. Reid, Amani M. Allen

**Affiliations:** 1 Department of Public Health and Recreation, College of Health and Human Sciences, San José State University, One Washington Square, San Jose, CA 95192-0052 USA; 2 School of Public Health, University of California, Berkeley, 2121 Berkeley Way, Room 5302, CA 94720-7360 Berkeley, USA; 3 Environmental Health Investigations Branch, California Department of Public Health, 850 Marina Bay Parkway, Building P, 3rd Floor, Richmond, CA 94804-6403 USA; 4 College of Environmental Design, University of California, 230 Bauer Wurster Hall #1820, Berkeley, CA 94720-1820 USA

**Keywords:** Healthy Places Index, Neighborhood, Preterm birth, Black women, Place-based

## Abstract

Black women have the highest incidence of preterm birth (PTB). Upstream factors, including neighborhood context, may be key drivers of this increased risk. This study assessed the relationship between neighborhood quality, defined by the Healthy Places Index, and PTB among Black women who lived in Oakland, California, and gave birth between 2007 and 2011 (*N* = 5418 women, *N* = 107 census tracts). We found that, compared with those living in lower quality neighborhoods, women living in higher quality neighborhoods had 20–38% lower risk of PTB, independent of confounders. Findings have implications for place-based research and interventions to address racial inequities in PTB.

**Supplementary Information:**

The online version contains supplementary material available at 10.1007/s11524-022-00624-8.

## Introduction

Preterm birth (PTB)—birth before 37 weeks of gestation—is a leading cause of infant mortality and risk factor for adverse health outcomes disproportionately experienced by Non-Hispanic Black and African American birthing women^1^ in the United States (U.S.) (hereafter, Black women) [1–5]. In 2019, Black women experienced 1.55 times the incidence of PTB (14.39%) compared with that of Non-Hispanic White birthing people (hereafter, White women) (9.26%) [6]. This inequity has persisted for decades, largely unexplained by individual risk factors such as maternal age, socioeconomic status, health status, interpregnancy interval, and parity [2, 6–10]. Building on a history of neighborhood effects research, studies are examining the neighborhood environment in which Black women live as a context for risk, protection, and intervention related to PTB inequity.

There is an array of evidence that physical, service, and social environments of a birthing person’s neighborhood may impact their risk of PTB [11, 12]. With respect to the physical environment (the quality of the natural and built environments of a neighborhood), for example, studies have found relationships between PTB risk and increased exposure to air pollution [13–16], proximity to waste facilities [17], retirement of coal and oil powerplants [18], and community green space [17]. Studies have also identified relationships between PTB and a neighborhood’s service environment (the presence of services and resources), such as the presence of and proximity to convenience stores, supermarkets, and grocery stores [19]. Additionally, studies have identified relationships between PTB and the social environment (including how neighbors interact with each other, perceive safety, and feel supported and connected), such as between community violence and PTB [20–24]. Many studies have also documented relationships between neighborhood socioeconomic deprivation (sometimes seen as part of the social, physical, or service environments, other times seen as characterizing neighborhood quality more broadly) and PTB [25–33].

The exposure to and experiences of harmful neighborhood characteristics may be distinct for Black women, contributing to the observed inequity in risk of PTB [11, 34, 35]. Neighborhood environments in the U.S. are shaped by the historical and contemporary realities of structural racism—intersecting and mutually reinforcing societal systems and institutions which foster and perpetuate racial discrimination and structure opportunities inequitably—in the U.S. [34, 36, 37]. Due to the country’s long history of racial residential segregation [37], Black women may be more likely to live in more impoverished and less-well-resourced neighborhoods than their White peers, regardless of individual socioeconomic status [11, 12, 21, 38, 39]. This increases the likelihood that the neighborhoods in which Black women live may be characterized by neighborhood risk factors that impact PTB (e.g., harmful pollutants, diminished accessibility of resources such as healthcare, and chronic stressors such as limited job or service opportunities, safety concerns, or over-policing) [11, 12, 38, 40–42]. In addition, studies that stratify results between Black women and White women have found different relationships between the same neighborhood characteristics and PTB for the two groups [14, 30, 43–45]. This suggests that the experience of the same neighborhood characteristics may be meaningfully different for Black women as compared with White women [35]. This too is a result of structural racism and the multiple pathways through which Black individuals experience racism [11, 34]; for example, targeted policing experienced by Black individuals living in a predominantly White neighborhood [46]. The discipline of Black Geographies describes the inextricable links between racism, capitalism, and the construction and experiences of place for Black communities [47]. Ultimately, as described by Frohlich and Potvin, in order to address health inequities, it is necessary to take a “vulnerable populations approach” in which research and intervention development is tailored to and developed in collaboration with those who have an inequitably heighted “risk of risks” for a given health outcome [48]. Therefore, to further understand how neighborhood quality may contribute to racial inequities in PTB in support of targeted intervention development, it is important to conduct this research among Black women.

In addition, to conduct effective research on the relationship between neighborhood quality and PTB among Black women, it is necessary to consider neighborhood quality as a multi-dimensional construct. In an attempt to isolate modifiable neighborhood characteristics, studies of neighborhood quality and PTB often examine individual neighborhood characteristics [49] or include multiple neighborhood variables in a single model to assess their independent effects [50]. These approaches mask the complexity of neighborhood environments in which physical, social, and service characteristics influence and are influenced by each other [51]. Furthermore, because structural racism shapes multiple dimensions of neighborhoods simultaneously (e.g., access to credit, the placement of industrial plants and highways, accessibility of grocery stores, and policing), focusing on individual neighborhood characteristics may not fully capture the overlapping and additive impact of multiple neighborhood factors on Black women’s risk of PTB [34, 37, 38, 51–54]. Rather than focusing on a single element of a neighborhood environment, practitioners of place-based community development emphasize the need to understand and intervene on multiple sectors simultaneously (i.e., housing, education, economic development, transportation, social service agencies) because of the ways in which these sectors interact with and affect each other [34, 55–57]. Therefore, defining neighborhood quality as a combination of physical, service, and social environmental factors has the potential to more precisely and comprehensively characterize a neighborhood. Additional research which considers the multidimensional nature of a healthy community is needed to inform place-based community development and support a long-term improvements in racial inequities in birth outcomes [51, 55–57].

This study examined the relationship between a multi-dimensional measure of neighborhood quality and risk of PTB among Black women in the city of Oakland, California. Oakland is a diverse urban environment in Northern California with over 400,000 residents, of whom 23% identify as Black or African American [58]. We focused on Oakland for three reasons. First, like many cities in the U.S., Oakland is home to racial inequities in PTB—11.7% for Black women as compared with 6.5% for White women [59]. There have also been significant investments in recent years in understanding and addressing racial inequities in PTB within Oakland specifically, with an emphasis on neighborhood transformation as an opportunity for intervention [57, 60–62]. In addition, ongoing place-based community development in Oakland could provide opportunities for evaluating the impact of transforming neighborhoods on racial inequities in preterm birth [63, 64]. By focusing this study in Oakland, our research aims to complement existing efforts and provide information to inform place-based community development moving forward. We hypothesized that living in an overall higher quality neighborhood would be associated with reduced risk of PTB among Black women in Oakland.

## Methods

### Study Population

The population for this retrospective cohort study was derived from all singleton births between 2007 and 2011 without congenital abnormalities and with recorded gestational age (between 20 and 44 weeks) on birth certificate or prenatal screening data to Non-Hispanic Black or African American women residing in an Oakland, California census tract (*N* = 6403 births). As part of the Life Course Social Context and Disparities in Birth Outcomes Study [31], data from California certificates of live birth from 2007 to 2011 linked (where available) to information from California’s statewide Prenatal Screening Program [65] were obtained from the California Biobank Program, and maternal residence at time of giving birth was geocoded to census tracts (the 2010 census tract vintage was used for this study).

Among women who had more than one birth between 2007 and 2011 (*N* = 608 women, average of two births per woman), one birth was randomly selected for inclusion (*N* = 669 births excluded). Women who resided in an Oakland census tract for which the exposure variable could not be calculated due to unavailable data were excluded (*N* = 64 women). Women with any missing covariates were also excluded (*N* = 252 women). The final study population included 5418 women and births. This study was approved by the Committee for the Protection of Human Subjects of the California Health and Human Services Agency (Project 14–01-1466), with IRB reliance approval from University of California, Berkeley.

### Outcome: Preterm Birth

Preterm birth (PTB) (< 37-week gestation) was coded as a dichotomous variable (1 = PTB, 0 = Term birth) based on the infant’s gestational age. Gestational age was defined using data from birth and prenatal screening records following a hierarchy of available data sources [31], with highest priority given to prenatal screening estimates where available (47.79% of study population births), largely derived from ultrasound [66] or high-quality last menstrual period dates [67]. When prenatal screening estimates were not available, gestational age was derived from birth records using obstetric estimate at time of delivery (51.99%) [68] or last menstrual period from birth records (0.22%).

### Exposure: Healthy Places Index

Neighborhood quality was based on the California Healthy Places Index (HPI), a publicly available, multi-dimensional characterization of census tracts in California developed in 2017 by the Public Health Alliance of Southern California, the Virginia Commonwealth University Center of Society and Health, and a 22-person steering committee of subject matter experts and health department representatives [69, 70]. The HPI score “capture[s] the additive influence of place-based domains on community health” [70] (p355) to quantify a California neighborhood’s potential to support residents’ health and wellbeing.

The HPI includes 25 indicators grouped into one of eight policy action domains—economics, education, healthcare access, housing, neighborhood conditions, pollution/clean environment, social environment, and transportation [70] (Supplementary Table I). Indicators for a given domain were standardized using *Z* scores (and multiplied by − 1 as needed to ensure that higher values are indicative of greater advantage) and averaged to form the domain score ($\overline{Z }$). Weighted quantile sum regression (using the WQS R-package) [71] was used to identify domain weights that maximize the ability for summed domain scores to predict life expectancy at birth (LEB), an accepted measure of the status of a community population’s health [69, 70, 72] A minimum weight of 0.05 was imposed to ensure that all domains, which had been identified as meaningful for policy action, were included. Finally, the weighted domain scores for a census tract were summed to create the HPI score.

Because the current HPI uses indicator data from 2005 to 2015, this study recreated the HPI for eligible Oakland neighborhoods during the 2007–2011 time period, following the developers’ methods for HPI construction (modified when necessary based on data availability) [69, 70] Permission to use and reproduce the HPI was granted by the Public Health Alliance of Southern California and the California Department of Public Health; however, use of the HPI does not imply endorsement by either entity [73]. Eligible census tracts were those with 2010 Census population estimates of ≥ 1500, a requirement for the original HPI construction. This resulted in the exclusion of five of the 113 Oakland census tracts (4%) and the 64 women residing within those census tracts. When indicator data for the 2007–2011 time period were unavailable, we included data from the next-closest time period (Supplementary Table I). The domain weights for the 2005–2014 Oakland HPI (hereafter referred to as “the Study HPI” or “the HPI”) were as follows: social environment (0.32), economic (0.21), neighborhood (0.14), education (0.10), transportation (0.07), healthcare (0.06), housing (0.06), and clean environment (0.05).

Spearman correlation coefficients were calculated for continuous HPI and domain scores across the 108 Oakland census tracts (Supplementary Table II). Though the HPI was significantly correlated with each component domains, no single domain was completely correlated with the HPI, demonstrating that the HPI is characterizing census tracts in a distinct manner. In analyses, the continuous HPI score was divided in to quartiles representing low-, mild-, moderate-, and high-quality neighborhood environments (higher HPI score = higher quality). The use of quartiles enhanced interpretability while allowing for assessment of variation across levels of neighborhood quality. In sensitivity analyses, the eight individual domain scores were also categorized into quartiles to assess whether any single domain might be driving observed relationships between HPI and risk of PTB among Black women in Oakland.

### Covariates: Maternal Characteristics

The following *a-priori* confounders, which could influence both where a woman lived at the time of giving birth and her likelihood of experiencing PTB, were included [13, 15, 23, 27, 74, 75]: age (continuous), parity (1 = first live birth, 0 = not first live birth), educational attainment-for-age (0 = low [≥ 15 with 8th grade or less, > 19 with 9–11 years of education], 1 = appropriate [13–14 with 8th grade or less, ≤ 19 with 9–11 years, > 17 with high school graduation], 2 = high [< 18 with high school graduation, any age with some college, college graduation, or more than college]) [76], nativity (1 = foreign-born, 0 = U.S. born), WIC benefits during pregnancy (1 = yes, 0 = no), and Medi-Cal benefits during pregnancy (1 = yes, 0 = no). All confounders were included in fully adjusted models.

### Statistical Analysis

After removing one census tract which included no study births, a total of 107 census tracts (94.69% of Oakland census tracts) were included in the study. We characterized the 107 study census tracts overall and across the HPI quartiles based on average 2007–2011 American Community Survey census tract population estimates, average number of study births per tract, and average number of study preterm births per tract. Maternal characteristics were described (number and frequency or mean, standard deviation, minimum, and maximum) for the total population, by PTB status, and by HPI quartile.

The relationship between the HPI and PTB among Black women residing in Oakland, California, at the time of giving birth (2007–2011) was assessed using multilevel log-linear generalized estimating equation (GEE) models [77] with exchangeable correlation structure to produce population average risk ratios (RRs) and 95% confidence intervals (CIs). Using a model sequence, we first assessed unadjusted associations (model 1), and then associations adjusted for all confounders (model 2). In a sensitivity analysis, we also assessed unadjusted and adjusted associations for each domain and PTB. Statistical analysis was conducted using SAS® software, Version 9.4 [78]. Copyright 2013, SAS Institute Inc. SAS, and all other SAS Institute Inc. product or service names are registered trademarks or trademarks of SAS Institute Inc., Cary, NC, USA.

## Results

Of the 107 study census tracts, 93 (86.9%) had at least one PTB. The average tract population size was 3596 (standard deviation (SD): 1217), with the largest average population size in the HPI-based low-quality tracts (Mean (M): 3780; SD: 1427). Across all tracts, there was an average of 51 births per tract (SD: 37) and 6 PTB per tract (SD: 5). The largest average number of study births (M: 83, SD: 38) and PTB (M: 9, SD: 5) were among the low-quality tracts. PTBs accounted for 10.2% of births experienced by Black women living in Oakland at the time of giving birth (2007–2011) (Table 1). Across the total study population, greater than 50% of women experienced a term birth, were multiparous, were U.S.-born, utilized WIC, or utilized Medi-Cal. Compared to women with term births, women with PTBs had a significantly higher proportion of multiparous women, U.S.-born women, and women with appropriate or low educational attainment for age; women with PTBs were also on average slightly but statistically significantly older than women who had term births (27.4 as compared to 26.6). Significant variation across women residing in neighborhoods within different HPI quartiles (low-, mild-, moderate-, and high-quality neighborhoods) was observed for birth outcome, nativity, age, educational attainment for age, WIC usage, and Medi-Cal usage. The study population living in the lowest HPI quartile neighborhoods were significantly younger and had significantly higher proportions of women who experienced PTB, U.S.-born women, women with low educational-attainment-for-age, women receiving WIC, and women receiving Medi-Cal compared with the study population living in other HPI quartile neighborhoods.

**Table 1:** Description of birth and maternal characteristics for the study population, by preterm birth status, and by Healthy Places Index (HPI) quartile

	Total	*Birth outcome*			*HPI quartiles (high HPI* = *high quality)*				
		Preterm	Term	*p value*	Low	Mild	Moderate	High	*p value*
Total population *N* (%)	5418 (100)	555 (10.2)	4863 (89.8)	–	2230 (41.2)	1565 (28.9)	1188 (21.9)	435 (8.0)	–
Birth outcome *N* (%)									
Preterm	555 (10.2)	–	–	–	256 (11.6)	163 (10.4)	105 (8.8)	31 (7.1)	0.01^c^
Term	4863 (89.8)	–	–		1974 (88.5)	1402 (89.6)	1083 (91.2)	404 (92.9)	
Parity *N* (%)									
First live birth	2619 (48.3)	245 (44.1)	2374 (48.8)	0.04^a^	1032 (46.3)	765 (48.9)	598 (50.3)	224 (51.5)	0.05^c^
Not first live birth	2799 (51.7)	310 (55.9)	2489 (51.2)		1198 (53.7)	800 (51.1)	590 (49.7)	211 (48.5)	
Nativity *N* (%)									
Foreign-born	448 (8.3)	33 (6.0)	415 (8.5)	0.03^a^	85 (3.8)	108 (6.9)	174 (14.7)	81 (18.6)	< 0.001^c^
U.S.-born	4970 (91.7)	522 (94.1)	4448 (91.5)		2145 (96.2)	1457 (93.1)	1014 (85.4)	354 (81.4)	
Age M (SD)	26.7 (6.5)	27.4 (6.9)	26.6 (6.5)	0.00^b^	25.7 (6.3)	26.1 (6.3)	27.6 (6.4)	31.1 (6.7)	< 0.001^d^
Education-for-age *N* (%)									
High	2617 (48.3)	224 (40.4)	2393 (49.2)	< 0.001^c^	880 (39.5)	726 (46.4)	681 (57.3)	330 (75.9)	< 0.001^c^
Appropriate	2181 (40.3)	254 (45.8)	1927 (39.6)		1036 (46.5)	654 (41.8)	406 (34.2)	85 (19.5)	
Low	620 (11.4)	77 (13.9)	543 (11.2)		314 (14.1)	185 (11.8)	101 (8.5)	20 (4.6)	
WIC *N* (%)									
Yes	3517 (64.9)	353 (63.6)	3164 (65.1)	0.51^a^	1631 (73.1)	1055 (67.4)	671 (56.5)	160 (36.8)	< 0.001^c^
No	1901 (35.1)	202 (36.4)	1699 (34.9)		599 (26.9)	510 (32.6)	517 (43.5)	275 (63.2)	
Medi-Cal *N* (%)									
Yes	3531 (65.2)	376 (67.8)	3155 (64.9)	0.19^a^	1646 (73.8)	1079 (69.0)	653 (55.0)	153 (35.2)	< 0.001^c^
No	1887 (34.8)	179 (32.3)	1708 (35.1)		584 (26.2)	486 (31.1)	535 (45.0)	282 (64.8)	

*N* (%), number (percentage); *M* (*SD*), mean (standard deviation); ^a^Fischer’s exact test; ^b^Pooled *t* test test; ^c^Chi-square tests; ^d^Kruskal-Walis test

In fully adjusted models, women living in neighborhoods with highest quality HPI score had a lower risk of PTB compared to those living in lowest quality (aRR: 0.62, 95% CI: 0.44, 0.87) and to a lesser extent, living in a moderate-quality neighborhood was also significantly associated with reduced risk (aRR: 0.80, 95% CI: 0.64, 1.00) (Table 2). Estimates follow a gradient pattern, in which living in an increasingly higher quality neighborhood was associated with an increasingly lower risk of PTB.

**Table 2:** Relationship between Healthy Places Index and preterm birth among Black women living in Oakland, California (2007–2011)

	Model 1			Model 2		
	Risk Ratio	95% confidence interval		Risk ratio	95% confidence interval	
Intercept	0.11	0.10	0.13	0.06	0.04	0.09
Healthy Places Index						
Quality						
Low quality	1.00			1.00		
Mild quality	0.91	0.75	1.10	0.93	0.77	1.13
Moderate quality	0.77	0.61	0.96	0.80	0.64	1.00
High quality	0.62	0.45	0.86	0.62	0.44	0.87
Parity						
First live birth				1.03	0.87	1.22
Not first live birth				1.00		
Nativity						
Foreign-born				0.67	0.49	0.91
U.S.-born				1.00		
Age				1.03	1.02	1.05
Education-for-age						
High				0.67	0.53	0.84
Appropriate				1.00		
Low				0.98	0.81	1.20
WIC						
Yes				0.84	0.68	1.03
No				1.00		
Medi-Cal						
Yes				1.13	0.90	1.40
No				1.00		

**Fig. 1:**
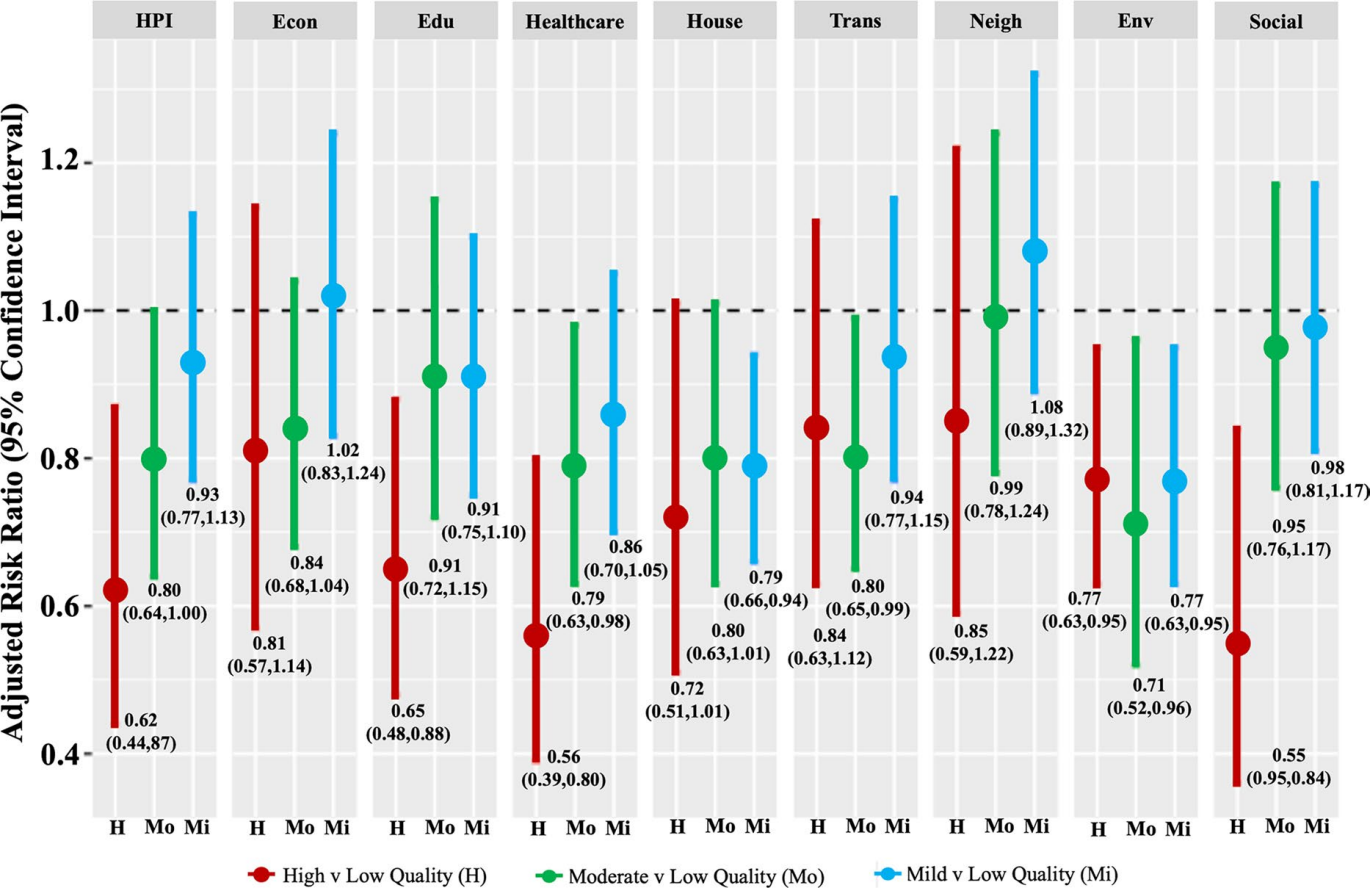
Comparison of adjusted risk ratios and 95% confidence intervals for HPI and eight component domains. Figure created in R and Microsoft PowerPoint

In the sensitivity analysis, significant independent protective relationships between living in a higher domain-specific-quality neighborhood compared to the lowest domain-specific-quality neighborhood were found for six of the eight domains (education, health, housing, transportation, clean environment, and social), with the strongest relationship in the social domain (aRR high-vs.-low quality: 0.55, 95% CI: 0.36, 0.84) across Oakland neighborhoods (Fig. 1). Adjusted risk ratios for five of the eight domains (economic, education, health, neighborhood, and social) followed a gradient relationship—compared to living in a low domain-specific quality neighborhood, living in an increasingly higher quality neighborhood was associated with an increasingly lower aRR.

## Conclusion

This study assessed whether living in a higher vs. low quality neighborhood, as defined by the HPI, was associated with reduced risk of PTB among Black women living in Oakland at the time of giving birth (2007–2011). PTB was experienced by 10% of the Black women in our study population. Across the Oakland census tracts, the risk of PTB was significantly reduced for those living in high- or moderate-quality neighborhoods vs. low-quality neighborhoods, independent of maternal characteristics. These results suggest that holistic neighborhood quality may be an important upstream risk factor to target for intervention to address the persistent racial inequity in PTB.

To our knowledge, this is the first published study to utilize the HPI to conceptualize neighborhood quality and its association with birth outcomes among Black women, and one of a few studies to consider the relationship between the HPI and any health outcome [79]. We chose the HPI because it represents neighborhood quality as a combination of a neighborhood’s physical, social, and service environments, all of which are thought to impact reproductive health outcomes [12] and continue to be shaped by structural racism in the U.S. [34, 37, 38, 51–54]. Scholars assessing the relationship between neighborhoods and birth outcomes tend to focus on the physical, social, or service environment in isolation [49] or include multiple dimensions of neighborhood environments as independent variables in a single model [50]. Neither of these approaches account for the fact that overall neighborhood quality cannot be defined based on a single dimension because these characteristics influence each other in distinct ways to form a neighborhood’s environment. We argue that in seeking to understand how neighborhoods may impact Black women’s risk of PTB, it is necessary to characterize neighborhood environments multidimensionally because of the complex ways in which structural racism simultaneously influences the physical, social, and service environments of a neighborhood [37, 54].

Multidimensional constructs of neighborhood quality, including dimensions of the physical, social, and service environments, have not often been used in studies assessing neighborhood effects on birth outcomes among Black women. In a mixed-methods study of infant mortality experienced by Black women from the National Birth Equity Collaborative, Wallace et al. [80] developed a Birth Equity Index that characterized metropolitan statistical areas (MSAs) across the country through a principal component analysis (PCA)-derived score of health, health behaviors, education, employment, racial segregation, pollution, racial income inequality, food access, crime rates, and jail admissions indicators. These indicators describe dimensions of physical, social, and service environments along with explicit measures of structural racism (e.g., racial segregation, racial income inequality). The authors found that worse overall neighborhood conditions were significantly associated with increased infant mortality rates among Black women. Our study results align with the implications of these findings—that overall lower quality neighborhood environments may increase risk among Black women for adverse birth outcomes—though we analyzed the PTB outcome in a single city, and the HPI is an asset-based measure at the smaller census tract level.

Neighborhood socioeconomic status (SES) is often used in birth outcomes research as a proxy for overall neighborhood quality and material deprivation [29, 30]. Neighborhood SES is typically an area-level aggregation of any one or a combination of individual-level SES measures [81] such as income or wealth, employment status or occupation, and educational attainment, sometimes combined with area-level housing and sociodemographic characteristics [28]. One widely used measure of neighborhood SES is the Neighborhood Deprivation Index (NDI), a summary measure of eight indicators: % of males in management and professional occupations, % of crowded housing, % of households in poverty, % of female-headed households with dependents, % of households on public assistance, % of households earning < $30,000 per year estimating poverty, % of adults earning less than a high school education, and % unemployed [28]. In using neighborhood SES in this way, an assumption is often made (explicitly or implicitly) that less wealth and opportunity in an area translates to fewer resources and services, greater physical and social disorder, and an increased proximity to harmful polluting industries or structures [28–30]. By not including measures of other neighborhood characteristics, however, neighborhood SES measures like the NDI may oversimplify the ways in which neighborhood characteristics cluster and intersect to impact health outcomes such as PTB.

The HPI includes most of the NDI indicators within its economic, education, housing, and social environment domains (Supplementary Table I) but extends the NDI through the inclusion of additional indicators in these domains as well as indicators of neighborhood conditions, healthcare access, clean environment, and transportation quality. The results of our sensitivity analysis demonstrate the importance of extending the NDI and other conceptualizations of SES to characterize neighborhood quality. In assessing whether any one domain might be driving a relationship between HPI and PTB, the sensitivity analysis found that multiple domains were significantly associated with PTB. Significant protective relationships against PTB were identified for women living in higher quality vs. low-quality neighborhood environments across the education, healthcare, housing, transportation, pollution/clean environment, and social domains. This suggests that the HPI is not describing the same neighborhood characterization as any individual domain and highlights the need to consider multidimensional neighborhood quality. Despite being correlated with each of the domains (Supplementary Table II), the economic domain was not significantly associated with PTB risk among Black women in our study. This aligns with the inconsistency of results from previous studies regarding the relationships between neighborhood SES and PTB risk among Black women [30, 32, 43, 44, 82, 83]. These findings underscore the fact that all low SES places are not created equal, making it necessary to move beyond solely using neighborhood SES when characterizing neighborhood quality. The HPI improves our conceptualization of how neighborhoods matter by incorporating characteristics beyond those of neighborhood SES.

Critics of using an overarching construct to characterize neighborhood quality emphasize the challenge of translating study findings into policy recommendations or community development practice without a single dimension of focus [43]. In this case, the HPI measure is uniquely positioned to be used by researchers and practitioners in cities across California. The HPI is publicly available and accessible to practitioners and community members through an online portal, ensuring broad access to the index for community-based work [84]. The domains and HPI score were derived from both literature and the experiences of practitioners and researchers from health departments around the state, suggesting that findings using these measures may be more directly translatable to local intervention and policy development. Because both the HPI and domain scores are available for assessment, scholars and practitioners have the opportunity to understand the particular combination of domain characteristics which contributed to a neighborhood’s overall HPI score, better facilitating targeted, multisector, place-based intervention. Local jurisdictions like Oakland could also follow the HPI process and work with a steering committee of local experts to adapt the HPI for incorporation of locally meaningful and available variables [69].

While we cannot interpret our results as causal, our findings suggest the need for further research into place-based community development interventions which seek to assess and intervene on multiple dimensions of neighborhood quality in order to have a long-term impact on racial inequities in PTB [51, 55–57]. Importantly, such research should focus on understanding Black women’s specific experiences of their neighborhood environments [85–88]. Structural racism shapes the neighborhoods in which Black families live, resulting in Black communities being systematically more likely to experience harmful and stressful physical, social, and service neighborhood environments, which may in turn increase Black women’s risk of PTB [21, 41, 85, 86, 88, 89]. Community development efforts to reduce racial inequities in PTB and other birth and maternal outcomes must center the experiences and priorities of Black women and their families.

The study findings should be considered in light of several limitations. Though the use of administrative census tract boundaries to define neighborhood is common, it is only a proxy for how neighborhoods may be defined by residents; analysis based on residents’ identified neighborhood boundaries or a relational conceptualization of place may yield different and more locally relevant results [51, 90]. We were also unable to assess how the quality of and movement through surrounding neighborhoods, both within and outside of Oakland, may have impacted a woman’s risk of preterm birth. Without considering the impact of daily exposures to neighborhoods of higher and/or lower quality, our estimates may under or overestimate the influence that neighborhood quality has on risk of PTB among Black women [91]. This is particularly salient for the city of Oakland, which is a part of the dynamic Bay Area region through which many residents move on a daily basis [92]. Future research seeking to understanding the influence of place on Black women’s risk of PTB should consider a more relational approach [90] to how participants construct and engage with place (as emphasized in the field of Black Geographies [47]).

Our decision to use the HPI—a strength because of its asset-based orientation, its development grounded in research and practice in California, and its public availability—also introduces important limitations. In following the structure of the HPI, we were not able to include other variables, such as neighborhood crime rates or locally defined characteristics, that are not available at a statewide level. Inclusion of such elements would likely increase the local relevance of a neighborhood quality measure, allowing for a more precise assessment of the relationship of interest. In addition, the HPI is an additive construct, and so does not model possible interaction across domains. As we saw in Supplementary Table II, there is significant correlation across domains, and use of an alternative approach to combining domains (e.g. principal component analysis) may characterize those relationships more effectively. Future research should compare analytic results based on the HPI with those of (a) a locally generated neighborhood construct and (b) an index based on a combination of HPI domains which considers interaction across domains in order to understand whether either alternative capture distinct elements of neighborhood quality.

Due to limitations of the data set, we were unable to account for the length of time in which women resided in their neighborhoods prior to becoming pregnant and giving birth, and thus cannot speak to how that variation might impact the effect of neighborhood quality on risk of PTB. Longer residence in one’s current neighborhood suggests a higher dosage of exposure to harmful and beneficial neighborhood qualities. Some research suggests that Black birthing persons are more likely to move to neighborhoods of greater deprivation in between pregnancies [93]. If this is the case for participants who are more recent residents of the Oakland neighborhoods included in this study, our estimates may in fact be conservative. These limitations highlight directions for future research—the use of locally meaningful neighborhood boundaries, assessing place from a relational perspective [90], and understanding how residential mobility and changes in neighborhood quality over time influence PTB.

Additionally, limitations of our data set prevented us from distinguishing between spontaneous and medically indicated preterm deliveries. Because the PTB is a complex outcome, and the etiologies of spontaneous and medically indicated preterm deliveries differ [94], our findings mask the potentially distinct relationships between neighborhood quality and each type of PTB [95]. Research allowing for this distinction is needed to better understand the mechanisms through which place-based intervention may impact PTB.

Finally, while our findings were always intended to be specific to Oakland, the unique nature of the study time period (during the Great Recession) calls into question whether the results are generalizable to present-day Oakland. Future research, which compares our findings with more recent Oakland birth data, would be beneficial for understanding the impact that the Great Recession may or may not have had on the relationship between neighborhood quality and PTB among Black women in Oakland.

The study’s findings suggest that living in a holistically defined, higher quality neighborhood may help to reduce Black women’s risk of experiencing PTB. For Oakland neighborhoods, the results identify domains for potential city-wide assessment and policy intervention to address the persistent racial inequities in PTB. Our results also highlight the importance of studying and intervening on multiple intersecting dimensions of neighborhood quality. Cities throughout California can access the publicly available HPI and its component domains to better understand the relationships between neighborhood quality and health outcomes like PTB in their own areas. Such efforts are needed to further research and practice to address health inequities affected by the places where we live.

## Supplementary Information

Below is the link to the electronic supplementary material.

Supplementary file1 (DOCX 18.3 KB)

Supplementary file2 (DOCX 16 KB)

## Footnotes

1 The terminology of “woman” is not inclusive of all birthing persons’ identities. All birthing persons whose birth certificates and prenatal screening data were included in this study were identified as “women.” We therefore use the language of “women” and “maternal” when describing the study participants.
