# Supplementary material for: Protective Places: the Relationship between Neighborhood Quality and Preterm Births to Black Women in Oakland, California (2007–2011)
Source: J Urban Health. 2022 Apr 6;99(3):492–505. doi: 10.1007/s11524-022-00624-8 (PMC9187821; doi:10.1007/s11524-022-00624-8)
Supplement: Supplementary file 1 — Supplementary file1 (DOCX 18.3 KB) [file 11524_2022_624_MOESM1_ESM.docx]

**Supplementary Table I: Study Healthy Places Index domains, indicators, data sources, and time periods used**

| **Domain** | **Indicator** | **Data Source** | **Time Period** |
| --- | --- | --- | --- |
| **Economic** | % of population with income > 200% of federal poverty line | Table S1701  American Community Survey | 2008-2012^a^ |
|  | % of population aged 25-64 who are employed | Table S2301  American Community Survey | 2007-2011 |
|  | Median household income | Table DP03  American Community Survey | 2007-2011 |
| **Education** | % of population over 25 with a bachelor’s education or higher | Table DP02  American Community Survey | 2007-2011 |
|  | % of 15-17-year-olds enrolled in school | Table S1401  American Community Survey | 2007-2011 |
|  | % of 3- and 4-year-olds enrolled in preschool | Table S1401  American Community Survey | 2007-2011 |
| **Healthcare Access** | % of adults ages 18-64 currently insured | Table S2701  American Community Survey | 2008-2012^a^ |
| **Housing** | % of occupied housing units occupied by property owners | Table DP04  American Community Survey | 2007-2011 |
|  | % of households with complete kitchen facilities and plumbing | Tables 15A, 15B, and 15C  Comprehensive Housing Affordability Strategy (CHAS) data | 2007-2011 |
|  | % of low-income homeowners paying more than 50% of income on housing costs | Healthy Places Index | 2007-2011 |
|  | % of low-income renter households paying more than 50% of income on housing costs | Healthy Places Index | 2007-2011 |
|  | % of households with < 1 occupant per room | Table DP04  American Community Survey | 2007-2011 |
| **Neighborhood Conditions** | % of population living within ½-mile of a park, beach, or open space great than 1 acre | California Health and Human Services Open Data platform | 2010 |
|  | Population-weighted % of census tract area with tree canopy | Healthy Places Index | 2011 |
|  | % of urban and small-town population resident less than ½ - mile from a supermarket/large grocery store, and percent of rural population living less than 1 mile from a supermarket/large grocery store | United States Department of Agriculture Food Access Research Atlas (archived) | 2010 |
|  | % of population residing within ¼ mile of an off-site sales alcohol outlet | Healthy Places Index | 2014^b^ |
|  | Combined employment density for retail, entertainment, and educational uses (jobs/acre) | Health Deprivation Index | 2010 |
| **Clean Environment** | Spatial distribution of gridded diesel PM emissions from on-road and non-road sources for a 2010 summer day in July (kg/day) – census tracts ordered by diesel PM concentration values and assigned a percentile based on the statewide distribution of values | Cal EnviroScreen 2.0 | 2010 |
|  | Cal EnviroScreen 2.0 drinking water contaminated index for selected contaminants | Cal EnviroScreen 2.0 | 2005-2013^c^ |
|  | Mean summer months (May-October) of the daily maximum 8-hour ozone concentration (ppm), averaged over three years (2012-2014) – census tracts were ordered by ozone concentration values and assigned a percentile based on statewide distribution of values | Healthy Places Index | 2012-2014^d^ |
|  | Annual mean concentration of PM2.5 (average quarterly means, mg/m^3^) over three years (2009-2011) – census tracts were ordered by PM2.5 concentration values and assigned a percentile based on the statewide distribution of values | Cal EnviroScreen 2.0 | 2009-2011 |
| **Social** | % of registered voters voting in the 2010 general election | Health Deprivation Index | 2010 |
|  | % of family households with children < 18 with two parents | Table B09008  American Community Survey | 2007-2011 |
| **Transportation** | % of households with access to an automobile | Table DP04  American Community Survey | 2007-2011 |
|  | % of workers (16 years +) commuting by walking, cycling, or transit (excluding working from home) | Table B08301  American Community Survey | 2007-2011 |
| **Life Expectancy at Birth** | Life expectancy at birth in 2010 | Healthy Places Index | 2010 |

1. *Data first made available in American Community Survey for 2008-2012 5-year estimates*
2. *Indicator constructed for HPI, methods for constructing indicator from raw data not made publicly available*
3. *Water contaminant compliance period used in Cal Enviroscreen 2.0*
4. *The ozone indicator from Cal EnviroScreen 3.0 (2012-2014) used in the HPI differs from that of Cal EnviroScreen 2.0 (covering an earlier time period), and so the HPI indicator was used.*

*Additional notes: Due to insufficient data, the 2007-2011 American Community Survey did not include estimates of % of 15-17-year-olds enrolled in school for five Oakland census tracts, estimates of % of 3- and 4-year-olds enrolled in preschool for one Oakland census tract, and estimates of either indicator for one Oakland census tract. Values of these indicators for these census tracts were imputed using a weighted average of its 10 nearest neighborhoods using the DMwR package in R (Torgo, 2010). The ozone indicator was excluded from the calculation of the Clean Environment domain because there was no variation across Oakland census tracts.*
