# Supplementary material for: Protective Places: the Relationship between Neighborhood Quality and Preterm Births to Black Women in Oakland, California (2007–2011)
Source: J Urban Health. 2022 Apr 6;99(3):492–505. doi: 10.1007/s11524-022-00624-8 (PMC9187821; doi:10.1007/s11524-022-00624-8)
Supplement: Supplementary file 2 — Supplementary file2 (DOCX 16 KB) [file 11524_2022_624_MOESM2_ESM.docx]

**Supplementary Table II: Spearman correlation coefficients among the Study Healthy Places Index and Component Domains across 108 Oakland Census tracts**

|  | **HPI** | **Economic** | **Education** | **Environment** | **Health** | **Housing** | **Neighborhood** | **Social** | **Transportation** |
| --- | --- | --- | --- | --- | --- | --- | --- | --- | --- |
| **HPI** |  |  |  |  |  |  |  |  |  |
|  |  |  |  |  |  |  |  |  |  |
| **Economic** | 0.91129 |  |  |  |  |  |  |  |  |
|  | <.0001* |  |  |  |  |  |  |  |  |
| **Education** | 0.81168 | 0.72866 |  |  |  |  |  |  |  |
|  | <.0001* | <.0001* |  |  |  |  |  |  |  |
| **Environment** | 0.29515 | 0.2534 | 0.37239 |  |  |  |  |  |  |
|  | 0.0019* | 0.0081* | <.0001* |  |  |  |  |  |  |
| **Health** | 0.80603 | 0.82146 | 0.66873 | 0.319 |  |  |  |  |  |
|  | <.0001* | <.0001* | <.0001* | 0.0008* |  |  |  |  |  |
| **Housing** | 0.7852 | 0.86225 | 0.70819 | 0.34117 | 0.79516 |  |  |  |  |
|  | <.0001* | <.0001* | <.0001* | 0.0003* | <.0001* |  |  |  |  |
| **Neighborhood** | 0.51289 | 0.36115 | 0.3325 | 0.13681 | 0.35301 | 0.27961 |  |  |  |
|  | <.0001* | 0.0001* | 0.0004* | 0.158 | 0.0002* | 0.0034* |  |  |  |
| **Social** | 0.92802 | 0.7766 | 0.69215 | 0.19158 | 0.64181 | 0.64099 | 0.44639 |  |  |
|  | <.0001* | <.0001* | <.0001* | 0.047* | <.0001* | <.0001* | <.0001* |  |  |
| **Transportation** | 0.34612 | 0.2876 | 0.32731 | 0.21248 | 0.302 | 0.19586 | 0.17702 | 0.25639 |  |
|  | 0.0002* | 0.0025* | 0.0005* | 0.0273* | 0.0015* | 0.0422* | 0.0668 | 0.0074* |  |
| * Statistically significant at p<0.05 | | | | | | | | | |
